# OPG/RANKL/RANK gene methylation among alcohol-induced femoral head necrosis in northern Chinese men

**DOI:** 10.1186/s13018-021-02356-y

**Published:** 2021-03-27

**Authors:** Tiantian Wang, Fei Wang, Tingting Liu, Menghu Sun, Feimeng An, Chang Liu, Ye Tian, Yuju Cao, Jianzhong Wang

**Affiliations:** 1 Inner Mongolia Medical University, Hohhot, 010050 Inner Mongolia China; 2 Department of Orthopedics and Traumatology, The Second Affiliated Hospital of Inner Mongolia Medical University, Hohhot, 010030 Inner Mongolia China; 3 China-Japan Union Hospital of Jilin University, Changchun, 130031 Jilin Province China; 4 Inner Mongolia Autonomous Region Hospital of Traditional Chinese Medicine, Hohhot, 010110 Inner Mongolia China; 5 Zhengzhou Traditional Chinese Medicine (TCM) Traumatology Hospital, Zhengzhou, 450016 Henan Province China

**Keywords:** Alcohol-induced osteonecrosis of the femoral head (ONFH), OPG/RANKL/RANK, Methylation, CpG islands

## Abstract

**Background and purpose:**

Alcohol-induced osteonecrosis of the femoral head (ONFH) is a complex and heterogeneous disease. Genetic factors and epigenetic modifications are one of the pathogenesis of the disease. However, the influence of epigenetic factors on the disease has not been systematically studied. Our research aims to determine the methylation changes of alcohol-induced ONFH.

**Methods:**

An analytical cross-sectional study of a Chinese male population (50 alcohol-induced ONFH patients and 50 controls). The EpiTYPER of the Sequenom MassARRAY platform was used to detect the DNA methylation status of 132 cytosine-phosphate-guanine (CpG) sites in the OPG/RANKL/RANK gene promoter region.

**Results:**

In the whole study group, the chi-square test was used to analyze the methylation rate between the two groups, and six CpG sites were found to be different, among which OPG1_CpG_2, OPG3_CpG_4, RANK1_CpG_6, RANK3_CpG_10, RANKL2_CpG_21, and RANKL2_CpG_46 in the case group were higher than those in the control group, while OPG4_CpG_2 was lower than that in the control group. The results showed that in patients with alcohol-induced ONFH, 146 CpG sites were examined for differences in methylation levels compared with healthy controls, 32 of which were not detected, and 23 of the remaining 114 sites showed differences in methylation levels compared with alcohol-induced ONFH patients. Receiver operator characteristic (ROC) curve analysis demonstrated the methylation levels of OPG/RANKL/RANK could efficiently predict the existence of alcohol-induced ONFH.

**Conclusion:**

Our study of Chinese men suggests that several CpG sites in the OPG/RANKL/RANK gene in peripheral blood leukocytes of patients with alcohol-induced ONFH are in an abnormal methylation state (hypermethylation tended to be more frequent).

## Introduction

The reports of alcohol-induced osteonecrosis of the femoral head (ONFH) at home and abroad are gradually increasing. Femoral head necrosis is a serious concomitant disease widely recognized in clinical practice with high disability rate and seriously affects the physical and mental health of patients. In China, long-term excessive alcohol intake is one of the common causes of non-traumatic femoral head necrosis [1]. The pathogenesis is currently unclear, and studies have shown that the genome changes in epigenetics are closely related to its occurrence and development, including genetic polymorphism and methylation changes.

Ethanol can destroy bone homeostasis, which is the root cause of femoral head necrosis. Ethanol can not only directly inhibit the proliferation and differentiation of bone-forming cells and promote their apoptosis, but also induce their precursor cells to tend to differentiate into adipocytes, and aggravate the imbalance of bone homeostasis. Studies have found that the proliferative activity of bone marrow mesenchymal stem cells (BMSC) derived from the proximal femoral shaft of patients with alcoholic femoral head necrosis decreased [2].

Bone tissue cells are one of the most active cells in the human body. Osteoblasts and osteoclasts in the human body maintain a stable relationship and maintain bone metabolism. In the process of human bone remodeling, osteoclasts are the “initiators” of bone remodeling, and osteoblasts are the “mediators” of bone remodeling. The nuclear factor-kB receptor raises the activator of NF ligand (RANKL) and is the key to the coupling of bone cell osteogenesis and bone resorption. In 1997, different researchers discovered new members of the tumor necrosis factor receptor and ligand superfamily: osteoprotegerin (OPG), RANKL, and receptor activator of NF-kB (RANK) [3]. Subsequently, a number of studies have confirmed that OPG, RANK, and RANKL can regulate the differentiation and development of osteoclasts and affect their functions. RANKL binds to RANK on the surface of osteoclasts to promote the differentiation and activation of osteoclasts and inhibit their apoptosis. Osteostatin prevents the binding of RANKL to RANK, thus preventing the activation of osteoclasts, inhibiting the function of osteoclasts, reducing bone absorption, and playing a negative regulatory role [4–6].

Long-term excessive alcohol intake can make the ratio of OPG/RANK/RANKL unbalanced, causing loss of bone mass and bone tissue metabolism disorders; it may also cause changes in the intima of the femoral head nourishing the artery vascular wall and accelerating the hardening of the vascular wall and accelerate the formation of blood clots; it can make a series of changes in the body’s immune system, and the OPG/RANK/RANKL system can also produce an immune response to accelerate the occurrence of femoral head necrosis.

The so-called epigenetics refers to the genetic information contained in the structure of chromosomes, not the DNA sequence. The main form of epigenetic information stored in mammalian cells is DNA methylation. DNA methylation is an epigenetic DNA modification catalyzed by DNA methyltransferase 1 (DNMT1). Gene expression can be epigenetically regulated by changes in DNA methylation, in particular, site-specific DNA methylation changes in cytosine-phosphate-guanine (CpG). The small islands surrounding the 5′-untranslated region (5′-UTR) of genes, including the hypomethylation and hypermethylation of genes, may be the key promoters of disease progression. Almost all DNA methylation occurs in the CpG doublet. The clustered CpG regions are called CpG islands, which are common in the promoter region of genes and are switches that regulate gene expression [7, 8].

DNA methylation changes under environmental stress and is gradually recognized as the cause and regulator of human disease. Research on the relationship between the level of methylation in CpG-rich regions and diseases has been ongoing. There are more and more experimental evidences about the potential diagnostic and therapeutic effects of methylation on tumor diseases and metabolic bone diseases. However, little is known about the DNA methylation status of OPG/RANKL/RANK genes in femoral head necrosis. Our aim was to study the changes in CpG island methylation of these genes in patients with alcohol-induced ONFH.

## Materials and methods

### Study participants

From 2018 to 2020, there were a total of 100 subjects including 50 consecutive recruited alcohol-induced ONFH and 50 healthy controls at the Second Affiliated Hospital of Inner Mongolia Medical University. The control is based on the physical examination enrollment group, without alcohol-induced ONFH or other related diseases. This study was approved by the Hospital Ethics Committee with informed consent of all participants.

### Inclusion and exclusion criteria

All patients were diagnosed with alcohol-induced ONFH in accordance with the relevant standards established by the International Bone Circulation Research Association. The patients in our study met the following criteria: age 20–70 years old, patients had an alcohol intake of pure ethanol 400 ml/week (320 g/week, any type of alcoholic beverage) for more than 6 months, and a diagnosis of ONFH within 1 year; The patients should meet the imaging criteria for the diagnosis and staging of alcohol-induced ONFH: X-rays showed extensive osteoporosis, increased subchondral bone density, or cystic changes, typically with crescent signs, changes in the normal structure of the bone trabeculae, collapse of the femoral head, and narrowing of the hip joint space. Patients who could not be completely diagnosed by X-ray examination were additised to MRI examination, showing peripheral low-signal area on T2WI in the initial stage of the disease with progressive progression of the disease, line-like low-signal area on T1WI in the anterior and upper femoral head bearing area, and crescent necrosis area with low or uneven signal around the sclerosis margin. On late T2WI, moderate or high signal intensity due to intracellular fluid exudation or articular fluid filling fracture line, and uneven and slightly low signal surrounding the fracture line are typical double line signs, or significant collapse of the femoral head due to osteonecrosis, cystic degeneration, or fracture, deformed acetabular sclerosis, and marginal osteophyte formation. The exclusion criteria are that patients should have direct trauma and other risk factors (such as a history of corticosteroid use, familial inherited diseases, and other major diseases). The healthy control group is defined according to the following criteria: no symptoms of hip joint disease and no history of thromboembolism. People with severe chronic diseases and other significant family genetic diseases will be excluded.

### DNA isolation

A major advantage of blood-based DNA methylation is that blood samples are readily available to investigate DNA methylation in patients with femoral head necrosis. Blood samples were taken in EDTA tubes and centrifuged at 2000 rpm for 10 min. Blood samples are stored at −80°C for future experiments. Genomic DNA was extracted from the whole blood of steroid-induced ONFH patients and healthy controls, using the GoldMag-Mini Purification Kit (GoldMag Co. Ltd. Xian City, China).

### Primer design and PCR amplification

We obtained the information of OPG/RANKL/RANK from the UCSC Genome database (http://genome.ucsc.edu). The PCR primers for OPG/RANKL/RANK amplicon sequences (Table 1) were designed with the online tool “epidesigner” (http://www.epidesigner.com).

**Table 1:** Primers

Gene		Sequence (5′→3′)
RANK1	L	aggaagagagAAGAAAAAGAGATAGTGGTTGTTGGT
	R	cagtaatacgactcactatagggagaaggctCAAATAATACCCAAACTCCCCTAAT
RANK2	L	aggaagagagGGTTTTGATGTTGTTATTTTTTTTAAATGT
	R	cagtaatacgactcactatagggagaaggctCCTTCCCTATAAAAACTTTCAAATTC
RANK3	L	aggaagagagATTTGAAAGTTTTTATAGGGAAGGG
	R	cagtaatacgactcactatagggagaaggctAAACACTTAATTAAACAACACCTAAAA
OPG1	L	aggaagagagTTTTTGTTGTTTTTTATAAAGTTAGTAGGA
	R	cagtaatacgactcactatagggagaaggctACTACTACCACCTAATCTCCCAACC
OPG2	L	aggaagagagGAAAGGTGTAAAGTTTGGTTTAGGA
	R	cagtaatacgactcactatagggagaaggctAAAAAACCAAATAACAACAACCTCC
OPG3	L	aggaagagagTTTTTTGTTTTTTTAGGGGTTAGAT
	R	cagtaatacgactcactatagggagaaggctTCCTAAACCAAACTTTACACCTTTC
OPG4	L	aggaagagagGGGGGTGTGTAGAAAGTTTTAGG
	R	cagtaatacgactcactatagggagaaggctAAAAACACAAACACAACAACTACCC
OPG5	L	aggaagagagGGGTAGTTGTTGTGTTTGTGTTTTT
	R	cagtaatacgactcactatagggagaaggctCCAATCAAACATTAATTAAAAAAATTCCT
RANKL1	L	aggaagagagTTTTTTTTGATTGTTGGGTGAGTT
	R	cagtaatacgactcactatagggagaaggctAATCTCTAAAAACCCTTCCTATCCA
RANKL2	L	aggaagagagTAGAGGTGGGAGTGGAAGAGGTAGTT
	R	cagtaatacgactcactatagggagaaggctATCCCCTAAAAAAATAACCACTCAC
RANKL3	L	aggaagagagGATTTTTTGGGAAGGTGGTTATTTA
	R	cagtaatacgactcactatagggagaaggctCCAACAAAAACTACACCAAATACCT
RANKL4	L	aggaagagagTGTTATTTTTAAGATGTAGAAATAGGGAT
	R	cagtaatacgactcactatagggagaaggctACTACCTCTTCCACTCCCACCTCTAA

### DNA methylation analysis

The MassARRAY Epityper DNA methylation quantitative technology was used to detect the methylation degree of OPG/RANKL/RANK gene promoter in each specimen. The MassARRAY Epityper DNA platform combines base-specific digestion (molecular cleavage) and MALDI-TOF detection principles to realize multiple CpG analysis and detection. Unmethylated cytosine (C) is converted to uracil (U), while methylated cytosine is not affected. Therefore, sulfite treatment will produce methylation-specific sequence changes then use the T7 promoter on the 5′ end. The primers are used for PCR amplification. After the product is subjected to a base-specific enzyme digestion reaction, the size and molecular weight of the DNA fragments depend on the base changes after sulfite treatment. Flight mass spectrometry can measure the molecular weight of each fragment. The supporting software EpiTYPER automatically reports the degree of methylation of each corresponding fragment [9].

### Statistical analysis

SPSS statistics 20.0 (SPSS, Chicago, IL) was used for statistical analysis of the data. For all statistical analyses, differences were considered to be statistically significant if the *p* value was less than 0.05.

The normality of the methylation horizontal distribution was assessed by the Kolmogorov-Smirnoff test. The chi-square test was used to analyze the normal distribution data, and non-normal distribution data were analyzed by the non-parametric test. multivariable unconditional logistic regression was used to determine the odds ratio (OR) and its 95% confidence interval (CI) for each individual CpG site, adjusted for age. Receiver operating characteristic (ROC) curve analysis was performed on the methylation ability of each CpG individual site in the case group and the control group, including calculation of the area under the ROC curve (AUC).

## Result

A total of 100 people participated in this study with 50 patients and 50 healthy controls. The mean ages were 42.62 ± 10.46 years for the cases and 42.76 ± 11.19 years for the controls. The information of the alcohol-induced ONFH patients and healthy participants was shown in Table 2. There were no statistically significant differences in the mean age between cases and controls.

**Table 2:** Characteristics of the participants

Variables	Case (*n*=50)	Control (*n*=50)	*p* value
Sex (*N*)			
Female	0	0	
Male	50	50	
Age, years (mean ± SD)	42.62 ± 10.46	42.76 ± 11.19	0.949
Clinical stages			
Stage II	20		
Stage III	15		
Stage IV	15		

*p* ≤ 0.05 indicates statistical significance

*p* value was calculated by the independent samples *t* test

*SD* standard deviation

This research uses the MassARRAY EpiTYPER mass spectrometer analysis technology platform, which has the characteristics of high throughput, high accuracy, and high sensitivity. It can not only analyze multiple CpG sites of multiple genes, but also quantitatively detect each of the single genes, CpG units (with methylation rate to quantify the degree of methylation of each CpG unit). When the methylation value is greater than or equal to 0.05 in probability analysis, it is considered that methylation occurs at this site; when the methylation value is less than 0.05, it is considered that no methylation occurs. The relationship between OPG/RANKL/RANK gene methylation rate and risk of alcohol-induced ONFH is shown in Table 3. The chi-square test was used to analyze the methylation rate between the two groups, and seven CpG sites were found to be different, among which OPG1_CpG_2, OPG3_CpG_4, RANK1_CpG_6, RANK3_CpG_10, RANKL2_CpG_21, and RANKL2_CpG_46 in the case group were higher than those in the control group, while OPG4_CpG_2 was lower than that in the control group.

**Table 3:** Relationship between OPG/RANKL/RANK gene methylation rate and risk of alcohol-induced ONFH

Gene	Group	Number of subjects		*p* value
		Non-methylation	Methylation	
OPG1_CpG_2	Control	1	49	0.016*
	Case	9	41	
OPG3_CpG_4	Control	8	42	0.028*
	Case	17	31	
OPG4_CpG_2	Control	31	19	0.035*
	Case	20	29	
RANK1_CpG_6	Control	8	42	0.038*
	Case	17	33	
RANK3_CpG_10	Control	34	16	0.018*
	Case	43	6	
RANKL2_CpG_21	Control	25	25	0.011*
	Case	36	12	
RANKL2_CpG_46	Control	15	33	0.004**
	Case	29	19	

*p* was calculated by the chi-squared test

*p < 0.05

**p < 0.01

**Fig. 1:**
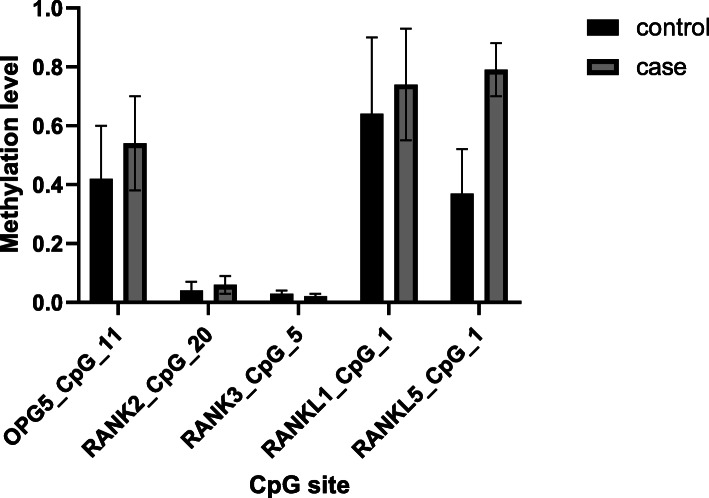
Methylation levels of OPG/RANKL/RANK genes

Our results showed that 146 CpG sites were measured, of which 32 were undetectable, and of the remaining 114 methylation sites, methylation levels were different in 23 CpG sites in patients with alcohol-induced femur head necrosis compared to healthy controls (Tables 4 and 5 and Fig. 1). The *t* test was used to test the methylation level between the cases and the control groups that did not meet the normal distribution (Table 5), and the non-parametric test was used to test the methylation level that did not meet the normal distribution (Table 4). Except that RANK3_CpG_5, OPG4_CpG_2, RANK1_CpG_33, RANK2_CpG_7.8, and RANK2_CpG_35 loci in the control group was higher than that in the case group, the methylation level of the other 18 sites was lower than that of patients with alcohol-induced ONFH.

**Table 4:** Methylation levels of OPG/RANKL/RANK genes

CpG site	Methylation levels		*Z*	*p*
	Case	Control		
OPG03_CpG_4	0.313	0.148	3.63	<0.001**
OPG04_CpG_2	0.087	0.093	2.29	0.022*
OPG04_CpG_13	0.011	0.011	2.01	0.045*
OPG05_CpG_1	0.207	0.148	2.52	0.012*
OPG05_CpG_2	0.207	0.158	2.06	0.039*
OPG05_CpG_4.5	0.154	0.107	3.23	0.001**
OPG05_CpG_8	0.095	0.082	2.10	0.036*
OPG05_CpG_10	0.276	0.227	2.93	0.003**
OPG05_CpG_11	0.539	0.421	3.46	<0.001**
RANK01_CpG_10.11	0.085	0.067	2.13	0.033*
RANK01_CpG_33	0.025	0.042	2.10	0.036*
RANK02_CpG_7.8	0.005	0.014	3.65	<0.001**
RANK02_CpG_16	0.089	0.072	4.11	<0.001**
RANK02_CpG_29	0.035	0.042	2.26	0.024**
RANK02_CpG_35	0.013	0.032	2.56	0.010*
RANK03_CpG_10	0.048	0.040	3.18	0.001**
RANKL_1_CpG_4	0.827	0.786	3.69	<0.001**
RANKL_2_CpG_46	0.168	0.100	2.35	0.019*

*p* was calculated by the non-parametric Wilcoxon signed rank test

*p < 0.05

**p < 0.01

**Table 5:** Methylation levels of OPG/RANKL/RANK genes

CpG site	Control (*N*)	Case (*N*)	Methylation level		*p*
			Control (mean ± SD)	Case (mean ± SD)	
OPG5_CpG_11	49	50	0.42 ± 0.18	0.54 ± 0.16	0.001**
RANK2_CpG_20	50	50	0.04 ± 0.03	0.06 ± 0.03	0.032*
RANK3_CpG_5	49	50	0.03 ± 0.01	0.02 ± 0.01	0.010*
RANKL1_CpG_1	49	48	0.64 ± 0.26	0.74 ± 0.19	0.033*
RANKL5_CpG_1	49	50	0.37 ± 0.15	0.79 ± 0.09	0.018*

*p* value was calculated by the independent samples *t* test

*p < 0.05

**p < 0.01

We further analyzed the correlation between methylation status and alcohol-induced femoral head necrosis by logistic regression (Table 6), which OPG3_CpG_5 (OR = 0.36, 95% CI 0.14–0.91, *p* = 0.031), RANK1_CpG_24.25.26.27 (OR = 0.13, 95% CI 0.03–0.62, *p* = 0.011), and RANK2_CpG_7.8 (OR = 0.15, 95% CI 0.04–0.56, *p*= 0.005) methylation shows reduced risk in alcohol-induced ONFH, and other 20 CpG site methylation increases the risk of alcohol-induced ONFH.

**Table 6:** Analysis of the relationship between OPG/RANKL/RANK methylation and ONFH risk induced by alcohol in men

Gene	OR	95% CI	*p*
OPG3_CpG_5	0.36	0.14–0.91	0.031*
OPG4_CpG_13	2.64	1.11–6.62	0.028*
OPG5_CpG_1	2.34	1.04–5.25	0.040*
OPG5_CpG_4.5	3.92	1.59–9.63	0.003**
OPG5_CpG_10	2.65	1.16–6.04	0.020*
OPG5_CpG_11	3.62	1.58–8.33	0.002**
RANK1_CpG_10.11	2.38	1.04–5.45	0.039*
RANK1_CpG_12.13.14	2.95	1.15–7.56	0.024*
RANK1_CpG_17	2.39	1.04–5.47	0.04*
RANK1_CpG_24.25.26.27	0.13	0.03–0.62	0.011*
RANK2_CpG_7.8	0.15	0.04–0.56	0.005**
RANK2_CpG_16	3.87	1.64–9.14	0.002**
RANK2_CpG_18	3.26	1.38–7.70	0.007**
RANK2_CpG_29	2.73	1.19–6.28	0.018*
RANK3_CpG_10	3.76	1.61–8.83	0.002**
RANKL1_CpG_4	3.12	1.31–7.46	0.010*
RANKL2_CpG_2	2.62	1.06–6.48	0.037*
RANKL2_CpG_12.13	3.01	1.31–6.92	0.009**
RANKL2_CpG_16.17	3.11	1.36–7.1	0.007**
RANKL2_CpG_21	3.55	1.50–8.41	0.004**
RANKL2_CpG_25.26.27	2.55	1.12–5.79	0.026*
RANKL2_CpG_46	3.39	1.45–7.90	0.005**
RANKL5_CpG_1	2.84	1.21–6..67	0.017*

*p* value adjusted for age was calculated by logistic regression

*OR* odds ratio, *CI* confidence interval

*p < 0.05

**p < 0.01

**Fig. 2:**
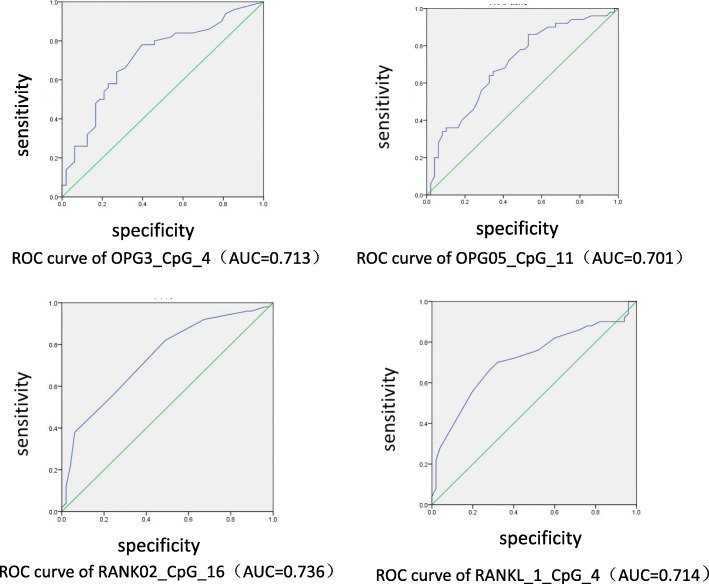
ROC curve of OPG/RANKL/RANK methylation level was used to analyze alcohol-induced ONFH

In order to evaluate the potential value of OPG/RANKL/RANK DNA methylation in alcoholic femoral head necrosis, we analyzed and evaluated all methylation sites and the average methylation level. ROC curves were plotted. The capacity of discrimination was assessed by calculating the AUC. In general, a useless test has an AUC of 0.5, while an ideal test (one with zero false negatives and zero false positives) has an AUC of 1.0. The maximum area under the curve value reached 0.736 (*p*<0.001), There were 4 CpG sites with an area under the curve value greater than 0.700, indicating that OPG/RANKL/RANK methylation has a higher predictive value in alcoholic femoral head necrosis (Fig. 2).

## Discussion

The onset of alcohol-induced ONFH is very complicated, and the exact mechanism is currently unclear, and there is a lack of effective prevention and treatment methods in the clinic [10]. Although in recent years, prosthesis replacement can finally solve the problem of patients’ activities, it also greatly increases the individual and social medical costs and the corresponding complications caused by prosthesis replacement. Some scholars believe that the onset of femoral head necrosis is a multifactorial and complex process. This means that in addition to environmental factors, genetic factors also play an important role in pathogenesis. However, to date, the DNA polymorphisms identified in GWAS explain less than 10% of the genetic risk, which indicates that the pathogenesis of these diseases also involves other factors, especially epigenetic mechanisms [11]. Epigenetics is a bridge connecting environmental factors and gene interaction. Environmental factors mainly regulate the transcriptional expression activity of target genes through histone modification mechanisms such as DNA methylation and acetylation, so as not to damage the integrity of the genome. Appropriate interpretation of the genomic information should be made to adapt to environmental changes [12].

OPG, RANK, and RANKL are closely related to bone metabolism. OPG can specifically bind to RANKL, inhibit osteoclast precursor cell differentiation and bone resorption of mature osteoclasts, and induce osteoclast apoptosis, thereby inhibiting osteoclast-mediated bone resorption. RANK is a receptor for RANKL. The specific binding of the two can stimulate osteoclast precursor cell differentiation, activate mature osteoclasts, prevent osteoclast apoptosis and prolong their lifespan, and promote bone resorption [13–15]. The relative concentration of RANKL and OPG in the bone is considered to be an important determinant of bone mass and strength, and inhibition of RANKL/RANK signaling has become a therapeutic target for diseases characterized by femoral head necrosis and other increased bone resorption [16, 17]. Epigenetic mechanisms are important for osteoclast differentiation [18]. Therefore, the study of OPG, RANK, and RANKL gene expression regulatory factors is very important for the study of bone metabolism.

Delgado-Calle et al. [19] found the methylation of RANKL and OPG genes in human bone tissue and confirmed that 5-aza-dC could inhibit the methylation of cell DNA, reduce the methylation of RANKL and OPG, and increase the expression of RANKL and OPG. Therefore, the methylation levels of OPG, RANK, and RANKL genes can reflect the protein synthesis level to a certain extent and play an important role in the regulation of bone metabolism balance. Our experimental results showed that in patients with alcoholic osteonecrosis, one site on OPG had a lower methylation level than the control group, and 9 sites showed hypermethylation; 4 sites on the RANK gene were hypomethylated, and 5 sites had higher methylation levels than the control group; 4 sites on RANKL were hypermethylated, and no positive results of hypomethylation were detected. Therefore, we speculated that the changes of OPG, RANK, and RANKL protein levels in patients with alcoholic femoral head necrosis might be related to the methylation level of related genes, which has certain guiding significance for the further study of epigenetic pathogenesis of femoral head necrosis.

DNA methylation is associated with transcriptional silencing of related genes. For example, Kitazawa and Kitazawa [20] studied mouse bone marrow stromal cell line and found that the differentiation of mesenchymal cells exists; RANKL gene methylation phenomenon, RANKL gene transcription start site around the CpG methylation, can silence gene promoter activity, thus inhibiting the expression of RANKL gene, regulating the differentiation of bone marrow stromal cell polymorphism, and influencing the osteoblast differentiation. Further experimental studies have shown that RANKL gene promoter TATA-box in the upper reaches of a single CpG locus of methylation can regulate cell and tissue specificity RANKL expression, thus affecting the osteoclast differentiation [21].

A growing body of research suggests that DNA methylation may be involved in the osteobiology of age-related diseases. In a recent study in China, 32 CpG sites in the RANKL promoter island were found to be highly demethylated in the osteoporosis group compared to healthy controls. The OPG promoter was hypomethylated in both cases and controls, but the levels were much higher in osteoporosis patients [22]. In a recent study, genome-wide DNA methylation was performed on the hip cartilage in ONFH patients. Furthermore, the accuracy of DNA methylation profile data and protein expression level of ONFH candidate differential methylated genes for cartilage injury of the hip joint was further verified [23].

In recent years, MSP and PCR product sequencing were mainly used to study the methylation of CpG sites at home and abroad. Only qualitative or limited methylation status of individual CpG islands could be detected, which greatly restricted the methylation analysis of CpG islands in the whole genome. In this study, we aimed to conduct a case-control study to study the methylation of the OPG/RANKL/RANK gene in patients with femoral head necrosis and healthy controls. The quantitative analysis method of peripheral blood DNA, that is, the MassARRAY EpiTYPER method, was used to find meaningful blood-borne femoral head necrosis early detection biomarkers. Through analysis of 50 patients with alcoholic osteonecrosis and 50 cases of healthy controls OPG/RANKL/RANK base 114 CpG loci of methylation status, think that these three gene methylation and methylation-specific loci low progress may be related to alcohol osteonecrosis has certain correlation, we found that in patients with alcoholic ONFH OPG/RANKL/RANK, high methylation of the gene is more common. However, it also provides evidence that DNA methylation may lead to ONFH. Therefore, the change of DNA methylation level in OPG/RANKL/RANK may be helpful for the detection of alcohol-induced ONFH.

The current research has certain limitations. Due to the relatively small number of participants, its statistical capabilities are limited. Retrospective or cross-sectional design does not allow us to determine that the observed correlation between changes in methylation status and femoral head necrosis is causal. In this study, the match between cases and age, gender, and race controls was insufficient to rule out potential confounding factors. Since gene expression is influenced and regulated by many factors in the process of translation, transcription, and protein synthesis, to assess the potential causal relationship between changes in CpG site methylation levels in the OPG/RANKL/RANK system and alcohol-induced ONFH, the role of DNA methylation in bone metabolism needs to be further studied and verified by large sample experiments.

## Conclusion

We performed the MassARRAY assay using peripheral blood DNA from alcohol-induced ONFH patients and healthy controls and found that methylation levels at some CpG sites in the peripheral blood differed between alcohol-induced ONFH cases and controls in OPG/RANKL/RANK, with more hypermethylation observed in alcohol-induced ONFH cases than in controls. Therefore, changes in DNA methylation levels in OPG/RANKL/RANK may be helpful for the detection of alcohol-induced ONFH.

## Data Availability

The data that support the findings of this study are available from the corresponding authors upon reasonable request.

## Abbreviations

ONFH: Osteonecrosis of the femoral head
CPG: Cytosine-phosphate-guanine
DNMT1: DNA methyltransferase 1
ROC: Receiver operator characteristic
AUC: Area under the curve
BMSC: Bone marrow mesenchymal stem cells
RANKL: Activator of NF ligand
OPG: Osteoprotegerin
RANK: Activator of NF-kB
